# Yinzhihuang oral liquid combined with phototherapy for neonatal jaundice: a systematic review and meta-analysis of randomized clinical trials

**DOI:** 10.1186/s12906-018-2290-x

**Published:** 2018-07-28

**Authors:** Ruo-han Wu, Shuo Feng, Mei Han, Patrina Caldwell, Shi-gang Liu, Jing Zhang, Jian-ping Liu

**Affiliations:** 1 Center for Evidence-based Chinese Medicine, Beijing University of Chinese Medicine, No. 11 North Sanhuan East Road, Chaoyang District, Beijing, 100029 China; 2 Beijing Hospital of Traditional Chinese Medicine, Capital Medical University, Beijing Institute of Traditional Chinese Medicine, No. 23 Gallery Back Street, Dongcheng District, Beijing, 100010 China; 3 Discipline of Paediatrics and Child Health, The Children’s Hospital at Westmead, University of Sydney, Locked Bag 4001, Westmead, NSW 2145 Australia; 4 Guang’anmen Hospital, China Academy of Chinese Medical Sciences, No. 5 Beixiange Street, Xicheng District, Beijing, 100053 China; 5 Department of Biostatistics and Epidemiology, Perelman School of Medicine, University of Pennsylvania, 3535 Market Street, Philadelphia, 19104 USA

**Keywords:** Neonatal jaundice, Yinzhihuang oral liquid, Systematic review, Meta-analysis

## Abstract

**Background:**

Neonatal jaundice affects at least 481,000 newborns every year. Phototherapy is recommended but it’s effects are limited and adverse reactions can occur. In China, phototherapy combined with Yinzhihuang oral liquid is also used for this condition. This systematic review evaluated the effectiveness and safety of combination therapy with Yinzhihuang oral liquid and phototherapy compared to phototherapy alone for treating neonatal jaundice.

**Method:**

A comprehensive literature search was performed in four Chinese databases, two English language databases and two trial registries from inception to June 2017. Two authors independently screened the citations and retrieved full publications for randomized trials on Yinzhihuang oral liquid combined with phototherapy for neonatal jaundice. The methodological quality of the trials was assessed according to the Cochrane Collaboration’s tool for assessing risk of bias. Data were analyzed using RevMan 5.3.

**Result:**

Totally 17 trials (involving 2561 neonates) were included in this review. Fourteen of them had a high risk of bias. Significant differences were detected between combination therapy and phototherapy alone for serum bilirubin level (MD − 50.25 μmol/L, 95% CI -64.01 to − 36.50, I^2^ = 98%; 7 trials, post-hoc decision choosing random effects model), failure of jaundice resolution (RR 0.21, 95% CI 0.14 to 0.32, I^2^ = 0%; 11 trials, fixed effects model), and time to jaundice resolution (MD − 2.17 days, 95%CI -2.96 to − 1.38, I^2^ = 98%; 6 trials, random effects model). Adverse events were reported in eight trials but none were serious. Trial sequential analysis for serum bilirubin level suggested that the cumulative Z-curve (which represents 1478 participants) reached the required information size (DARIS = 1301 participants).

**Conclusion:**

Based on trials with low methodological quality, Yinzhihuang oral liquid combined with phototherapy seemed to be safe and superior to phototherapy alone for reducing serum bilirubin in neonatal jaundice. These potential benefits need to be confirmed in future trials using rigorous methodology.

**Trial registration:**

Systematic review registration: [PROSPERO registration: CRD42016037691].

## Background

Neonatal jaundice, the most common condition that requires medical attention in newborns, presents as yellowish pigmentation of skin, mucous membranes or organs. The cause of neonatal jaundice is hyperbilirubinemia (defined as total serum bilirubin ≥171umol/L for premature or ≥ 256 umol/L for full term infants). Unconjugated bilirubin is a neurotoxin and excessive levels can cause kernicterus and bilirubin encephalopathy, which may result in devastating brain injuries and permanent neurodevelopmental damage. It is estimated that, worldwide, severe hyperbilirubinaemia affects at least 481,000 term or near term newborn babies annually, of whom 114,000 die and more than 63,000 survive with moderate or severe disability [1, 2].

The American Academy of Pediatrics(AAP) has provided guidelines for the management of neonatal jaundice [3]. AAP recommends that total bilirubin (TBil) and transcutaneous bilirubin (TCB) should be monitored when evaluating neonatal jaundice [4], and hyperbilirubinaemia above a certain threshold should be treated. The conventional therapies for this disease are phototherapy, exchange transfusion and albumin infusion. Phototherapy is a noninvasive, acceptable, safe and effective treatment [5]. Phototherapy has been used in clinical practice since the 1980s, when the National Institute of Child Health and Human Development reported it was as effective as exchange transfusion [6]. However, the light exposure from phototherapy may cause water loss, hypocalcemia, disorder of circadian rhythms, allergic diseases and melanocytic nevi [7]. Phototherapy may have limited effectiveness for high levels of serum bilirubin, which is associated with bilirubin encephalopathy and kernicterus.

In China, for infants that fail phototherapy monotherapy, combination therapy with phototherapy and Chinese herbal medicine is also used for this condition. In traditional Chinese medicine (TCM), the earliest record of jaundice was in the Inner Canon of Huangdi more than 2000 years ago, in which the detailed pathogenesis and symptoms were described*.* According to TCM theory, syndrome differentiation of this disorder often referred to retained dampness-heat stagnation, internal obstruction of cold-dampness and qi-blood stasis. Yinzhihuang oral liquid, original recorded in the TCM classical *Cold Damage and Golden Chamber*, is mainly composed of extracts of *Herba Artemisiae Scopariae*, *Fructus Gardeniae*, *Radix Scutellariae* and a small amount of *Flos Lonicierae Japonicae*. The effect of the oral liquid is attributed toremoving dampness to eliminate jaundice, and has been used in TCM to treat jaundice for over 1800 years [8]. Pharmacological studies revealed that the main components of this formula may inhibit hepatocyte apoptosis, promote the secretion and excretion of bile, as well as promote hepatic regeneration and prevent postoperative hepatic failure [9].

Previous clinical trials published in China indicated that the herbal medicine Yinzhihuang oral formula could reduce the bilirubin level in hyperbilirubinemia and facilitate jaundice resolution. However, the function and safety of Yinzhihuang oral formula in newborns is unknown. This systematic review aims to compare the effectiveness and safety of Yinzhihuang oral liquid used in combination with phototherapy with phototherapy alone on the treatment of neonatal jaundice.

## Methods

The present systematic review follows the PRISMA (Preferred Reporting Items for Systematic Review and Meta-analysis) guidelines and the handbook published by the Cochrane Collaboration.

### Eligibility criteria

#### Types of studies design

The present systematic review included randomized controlled trials (RCT), regardless of the methods of blinding, without restrict on language and setting.

#### Types of participants

Infants within 28 days of birth, with hyperbilirubinemia (a direct or conjugated bilirubin of more than17.1 μmol/L, or total serum bilirubin of more than 85 μmol/L and a direct bilirubin of more than 20% of the total serum bilirubin) or clinical features of visible jaundice in the sclera, body or limbs or other organs, regardless of the physiologic or pathologic cause of the jaundice, were included in this systematic review. The diagnostic criteria for neonatal jaundice followed international guidelines [3, 10].

#### Types of intervention

Yinzhihuang oral liquid combined with phototherapy (blue light) were included, without restriction on dosage or treatment duration.

#### Types of control

Phototherapy (blue light) was the control therapy in this systematic review.

#### Types of outcomes

##### Primary outcomes

Primary outcome measures were total serum bilirubin level (that the end of treatment, as tested by biochemical analysis) and serious adverse events [11].

##### Secondary outcomes

Secondary outcome measures were:

Failure of jaundice resolution, defined as the proportion of participants without significant resolution of yellow coloration in the skin and/or sclera after treatment, or without decline in total serum bilirubin, which was classified as failure of jaundice resolution.

Time to jaundice resolution defined as the duration from the commencement of treatment to jaundice resolution. This was according to the time recorded in the original studies. We acknowledged that this may introduce measurement bias, thus we did not select it as a primary outcome.

Any non-serious adverse events [11] data was also collected.

### Search strategy

#### Electronic searches

The following four Chinese language electronic databases, two English language electronic databases, and also two English trial registration platforms were searched from inception to June 2017: China Network Knowledge Infrastructure (CNKI) (1979–2017), Chinese Scientific Journal Database (VIP) (1989–2017), Wan Fang Database (1985–2017), Chinese Biomedical Literature Database (Sino-Med) (1978–2017), Medline (1966–2017), the Cochrane Library (2017, Issue 12), ClinicalTrials.gov (December 2017) and The World Health Organization Clinical Trials Registry Platform (WHO ICTRP) (December 2017). We utilized the medical subject headings “Yinzhihuang” or “Yinchenhao decoction” or “Herba Artemisiae Scopariae” and “neonatal jaundice” or “hyperbilirubinemia in the newborn infant” in both Chinese and English databases. Specific search strategies can be seen in Appendix.

#### Additional searches

Additional sources of studies included reference lists from previous systematic reviews on the topics of Yinzhihuang oral liquid and neonatal jaundice.

### Studies screening and selection

Only studies that assessed the use of Yinzhihuang Oral Liquid in combination with phototherapy for treating hyperbilirubinaemia in infants were included in the review.

Two authors (Wu RH and Han M) independently screened the literature for eligibility of trials, according to the criteria demonstrated above. Any inconsistency in this process was resolved by the third person (Liu JP).

### Data extraction

Two authors(Wu RH and Han M) independently extracted the following information: study characteristics including trial design, sample size, length of follow-up, method for statistical analysis, inclusion/exclusion criteria, baseline characteristics of participants, interventions, treatment duration, outcome measures, numbers of participants randomized, treatment completed, withdrawal/drop-out and loss to follow up, primary outcome (Total serum bilirubin), secondary outcome (rate of failure of jaundice resolution and time to jaundice resolution), and adverse events.

### Risk of bias in individual studies

Two authors (Wu RH and Han M) used the Cochrane Collaboration’s ‘Risk of Bias’ tool [12] to assess the methodological quality of the included studies. Seven items including random sequence generation, allocation concealment, blinding of participants and personnel, blinding of outcome assessment, incomplete outcome data, selective reporting and other bias were assessed as ‘low risk’, ‘high risk’, or ‘unclear risk’. Any disagreements were resolved by discussion with a third author (Liu JP).

### Synthesis of results

Meta-analysis was performed using RevMan 5.3. Risk ratios (RR) and mean differences (MD and their 95% confidence intervals (CI)were calculated for dichotomous or continuous data, respectively. Intention-to-treat (ITT) analysis was conducted. Heterogeneity was calculated using I-squared statistics. When heterogeneity inspection result showed significant heterogeneity (I-squared< 75%), we used random effects model (REM), otherwise we applied fixed effects model (FEM) [13]. For the primary outcomes, given that the included trials had differences in study population and intervention, we used REM as the primary analysis and FEM as the sensitivity analysis. This was a post-hoc choice for statistical analysis.

### Trial sequential analysis

To calculate the required information size in a meta-analysis to detect the robustness of the results, we conducted a trial sequential analysis [14, 15] by using software from The Copenhagen Trial Unit [14]. In our meta-analysis, the diversity-adjusted required information size was based on the proportion of events in the phototherapy group; with the assumption of a plausible RR reduction observed in the included trials with a relatively low risk of bias; a risk of type I error of 5%; a risk of type II error of 20%; and an assumed diversity of the meta-analysis [16]. If the trial sequential monitoring boundary for benefit or harm are crossed before the required information size is reached, it suggests that firm evidence has been established and further trials are unnecessary. On the other hand, if the boundary has not been reached, it suggests the need to continue doing trials in order to detect or reject a certain intervention effect.

### Sensitivity analysis

The sensitivity analysis was planned to detect the influence of missing data for primary outcomes. For continuous variables (total serum bilirubin level and time to jaundice resolution), if missing data were reported in original studies, we would fill the missing data with the mean in a continuous meta-analysis. For the binary variable (failure of jaundice resolution), if missing data were reported in original studies, we would perform best-worst scenario (assuming that participants with an unknown status of achieving jaundice resolution after receiving Yinzhihuang oral liquid did achieve jaundice resolution, and that all participants from the phototherapy group with an unknown status of achieving jaundice resolution did not achieve jaundice resolution) and worst-best scenario (assuming that participants with an unknown status of achieving jaundice resolution after receiving Yinzhihuang oral liquid did not achieve jaundice resolution and that all participants from phototherapy group with an unknown status of achieving jaundice resolution achieved jaundice resolution) in a binary meta-analysis [12].

### Subgroup analysis

We performed subgroup analysis according to the duration of treatment and dosage of Yinzhihuang oral liquid.

### Publication bias

Funnel plots [17, 18] were applied to detect publication bias if there were more than ten trials included in a meta-analysis.

### Assessment of quality of evidence

The GRADE software was used to generate ‘Summary of findings’ tables and to assess the quality of the body of evidence.

## Results

### General description of overall studies

**Fig. 1:**
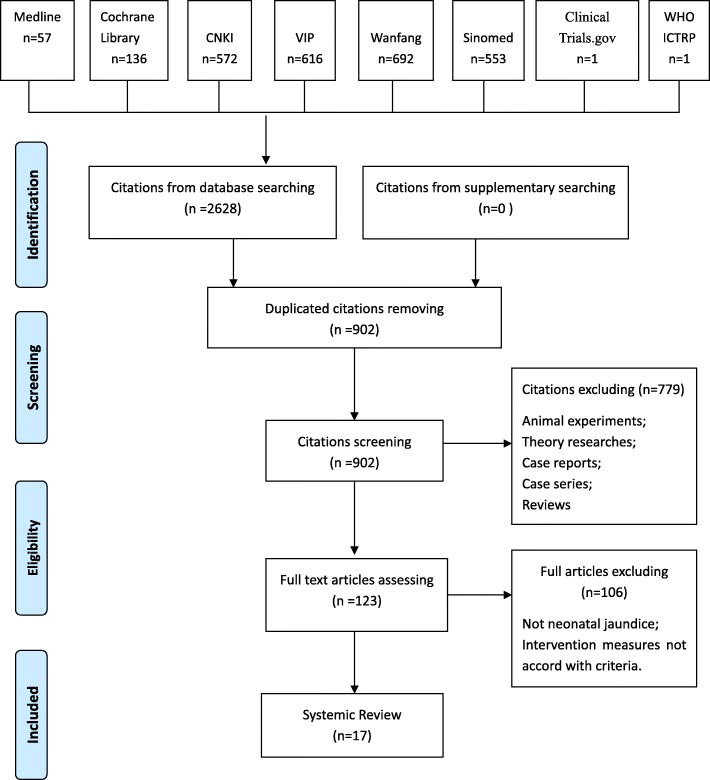
Flow chart of study selection

We initially identified 2628 citations. 902 articles remained after removal of duplications. After screening for eligibility by reading the title and abstract,779 articles were excluded and 123 full-texts were assessed further, with exclusion of another 106 articles. Finally,17 studies met the inclusion criteria (Fig. 1) and were included in systematic review.

These 17 included trials, which involved2561 neonatal jaundice participants, were all conducted in mainland China. They were published from 2004 to 2015. All had a parallel design with two arms. All the participants in the intervention arms had Yinzhihuang oral liquid combined with phototherapy, and were compared with phototherapy alone in control arms. In 5 trials [19, 24, 31, 32, 34], the herbal medicine was given at 3mls three times daily; In 8 trials [20–23, 26, 27, 29, 35], it was given at 5 ml twice daily; while 2 trials used 5 ml three times a day [28, 30]. Seven trials assessed total serum bilirubin levels and 11 trials assessed jaundice resolution after treatment. The characteristics of the included studies are summarized in Table 1.

**Table 1:** Characteristics of included trials

N	Study ID	Sample(Treatment/Control/Total)	diagnostic criteria	Intervention	Dosage and course duration	Control	Endpoints
1	Fu KG 2013 [19]	60/60/120	NA	Yinzhihuang oral liquid + Phototherapy	3 ml Po Tid	Phototherapy:Blue light; 8-12 h/d; 5d	Failure of jaundice resolution; Serious adverse events
2	Huang J 2013 [20]	58/56/114	Practical neonatology	Yinzhihuang + Phototherapy	5 ml Po Bid	Phototherapy:Blue light; 6-8 h/d; 3d	Failure of jaundice resolution; Total serum bilirubin
3	Huang N 2004 [21]	32/31/63	NA	Yinzhihuang + Phototherapy	5 ml Po Bid	Phototherapy	Span of jaundice resolution; Serious adverse events
4	Liang LL 2014 [22]	60/60/120	NA	Yinzhihuang + Phototherapy	5 ml Po Bid	Phototherapy:Blue light; 6-8 h/d; 3d	Failure of jaundice resolution; Total serum bilirubin
5	Li HF 2013 [23]	30/30/60	Chinese Internal Medicine	Yinzhihuang + Phototherapy	5 ml Po Bid	Phototherapy:Blue light; 16 h/d; 14d	Failure of jaundice resolution; Total serum bilirubin; Time to jaundice resolution; Serious adverse events
6	Li Q 2013 [24]	54/54/108	NA	Yinzhihuang + Phototherapy	3 ml Po Tid	Phototherapy:Blue light; 8-12 h/d	Time to jaundice resolution; Total serum bilirubin
7	Liu GH 2013 [25]	25/25/50	Practical neonatology	Yinzhihuang + Phototherapy	NA	Phototherapy:Blue light; 6d	Failure of jaundice resolution
8	Lu J 2014 [26]	42/42/84	practical paidonosology	Yinzhihuang + Phototherapy	5 ml Po Bid	Phototherapy:Blue light; 10 h/d; 7d	Failure of jaundice resolution; Total serum bilirubin; Time to jaundice resolution
9	Mu J 2015 [27]	43/43/86	Pediatrics	Yinzhihuang + Phototherapy	5 ml Po Bid	Phototherapy:Blue light; 5-7d	Failure of jaundice resolution; Total serum bilirubin; Serious adverse events
10	Qiu J 2014 [28]	60/60/120	Pediatrics	Yinzhihuang + Phototherapy	5 ml Po Tid	Phototherapy:Blue light; 5d	Failure of jaundice resolution; Total serum bilirubin
11	Sun ZY 2013 [29]	100/100/200	Expert Consensus Documents	Yinzhihuang + Phototherapy	5 ml Po Bid	Phototherapy:Blue light; 6 h Bid	Total serum bilirubin; Time to jaundice resolution; Serious adverse events
12	Wang DF 2014 [30]	100/100/200	NA	Yinzhihuang + Phototherapy	5 ml Po Tid	Phototherapy:Blue light; 12 h/d; 5d	Total serum bilirubin
13	Wu Y 2014 [31]	38/38/76	Practical neonatology	Yinzhihuang + Phototherapy	3 ml Po Tid	Phototherapy:Blue light; 8-12 h/d; 3-5d	Failure of jaundice resolution; Total serum bilirubin; Time to jaundice resolution; Serious adverse events
14	Xing XW 2012 [32]	54/54/108	Practical neonatology	Yinzhihuang + Phototherapy	3 ml Po Tid	Phototherapy:Blue light; 8-12 h/d; 5d	Failure of jaundice resolution; Total serum bilirubin; Time to jaundice resolution; Serious adverse events
15	Yang ZM 2013 [33]	49/49/98	NA	Yinzhihuang + Phototherapy	1 ml/kg Po Tid	Phototherapy:Blue light; 5d	Time to jaundice resolution; Serious adverse events
16	Zhao HY 2013 [34]	75/75/150	academic conference recommendation	Yinzhihuang + Phototherapy	3 ml Po Tid	Phototherapy:Blue light; 12 h/d; 7d	Failure of jaundice resolution; Total serum bilirubin
17	Clinical Research Collaborative Group of YZH 2011 [35]	395/409/804	AAP guideline	Yinzhihuang + Phototherapy	5 ml Po Bid	Phototherapy:Blue light; 8-12 h/d; 7d	Total serum bilirubin; Serious adverse events

Note: *NA* Not applicable, *Po* peroral, *Tid* three times a day, *Bid* twice a day

### Assessment of risk of bias

**Fig. 2:**
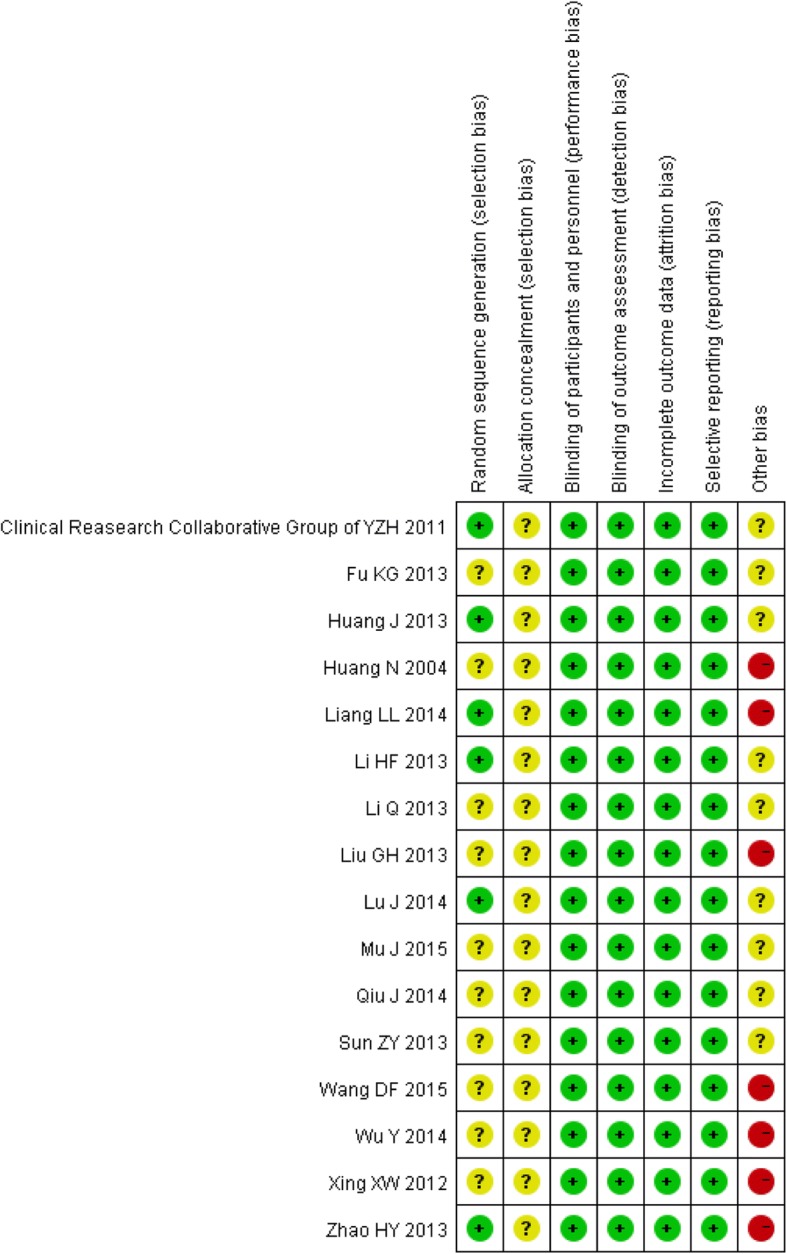
Risk of bias summary. Note: In each item, the color of green represents low risk of bias, the yellow represents unclear risk of bias, while the red represents high risk of bias

Overall, the methodological quality of included studies was low (Fig. 2). Most of the included studies did not specify the sequence generation process. No included studies report allocation concealment. Blinding was all assessed as low risk, as the participants were neonates and blinding would not have affected the objective measurement of serum bilirubin. All studies except one had low attrition bias as all participants were accounted for. For reporting bias, due to unavailability of protocols of any included trials, we had to check the methods and results sections, with all trials reporting according to what was described in methods section. All included studies claimed baseline comparability, and seven of these 17 studies did not report the inclusion/exclusion criteria for recruitment. None of the included studies appeared to have a high risk of for-profit bias as none were sponsored by pharmaceutical companies.

### Effects of intervention

#### Primary outcome

##### Total serum bilirubin

**Fig. 3:**
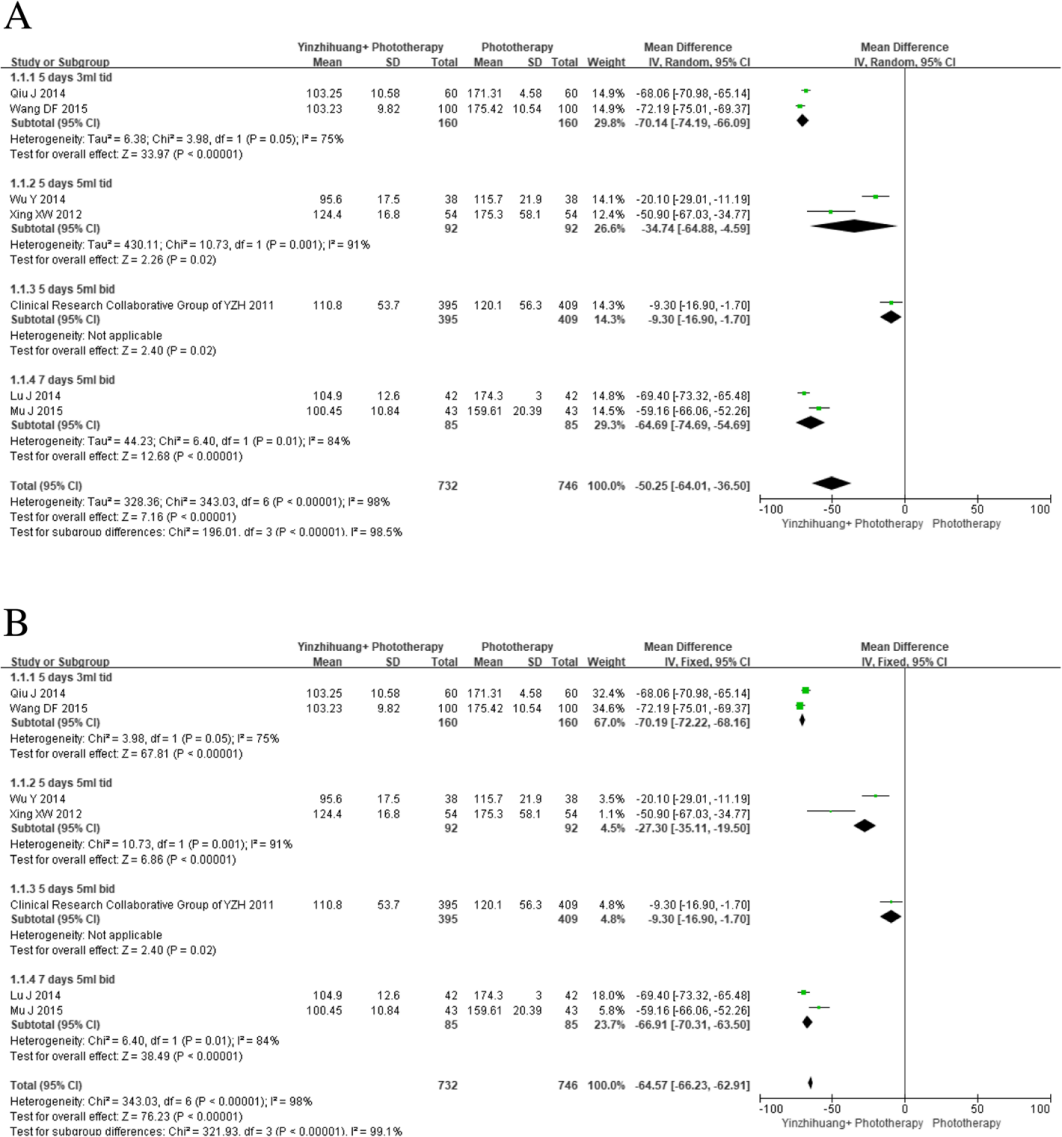
**a**. Forest plot of total serum bilirubin (μmol/l) and subgroup analysis based on dosage of Yinzhihuang oral liquid (random effect model). **b**. Forest plot of total serum bilirubin (μmol/l) and subgroup analysis based on dosage of Yinzhihuang oral liquid (fixed effects model)

Seven trials provided information on total serum bilirubin. Given the differences in dosage and course duration, REM was used for primary analysis. Being different from determination by I^2^ value, which documented in the study protocol, this is a post-hoc decision. Meta-analysis showed Yinzhihuang oral liquid combined with phototherapy was superior to phototherapy alone for reducing serum bilirubin (MD − 50.25 μmol/L, 95% CI -64.01 to − 36.50, I^2^ = 98%; seven trials, *n* = 1478, REM). In sensitivity analysis, FEM showed a larger effect size (MD − 64.57 μmol/L, 95% CI -66.23 to − 62.91, I^2^ = 98%; seven trials, n = 1478, FEM) (Fig. 3(a) and (b)).

Subgroup analysis based on dosage also revealed that combination therapy was better than phototherapy alone for all doses: for 5 days 3 ml Tid (MD − 70.14 μmol/L, 95% CI-74.19 to − 66.09, I^2^ = 75%; two trials, *n* = 320, REM) (MD -70.19 μmol/L, 95% CI -72.22 to − 68.16, I^2^ = 75%; two trials, n = 320, FEM), 5 days 5 ml Tid (MD − 34.74 μmol/L, 95% CI -64.88 to − 4.59, I^2^ = 91%; two trials, *n* = 184, REM) (MD -27.30 μmol/L, 95% CI -35.11 to − 19.50, I^2^ = 91%; two trials, n = 184, FEM), 5 days 5 ml Bid (MD − 9.30 μmol/L, 95% CI -16.90 to − 1.7; one trial, *n* = 804) and 7 days 5 ml Bid (MD − 64.69 μmol/L, 95% CI -74.69 to − 54.69, I^2^ = 84%; two trials, *n* = 170, REM) (MD -66.91 μmol/L, 95% CI -70.31 to − 63.50, I^2^ = 84%; two trials, n = 170, FEM) (Fig. 3(a) and (b)).

**Fig. 4:**
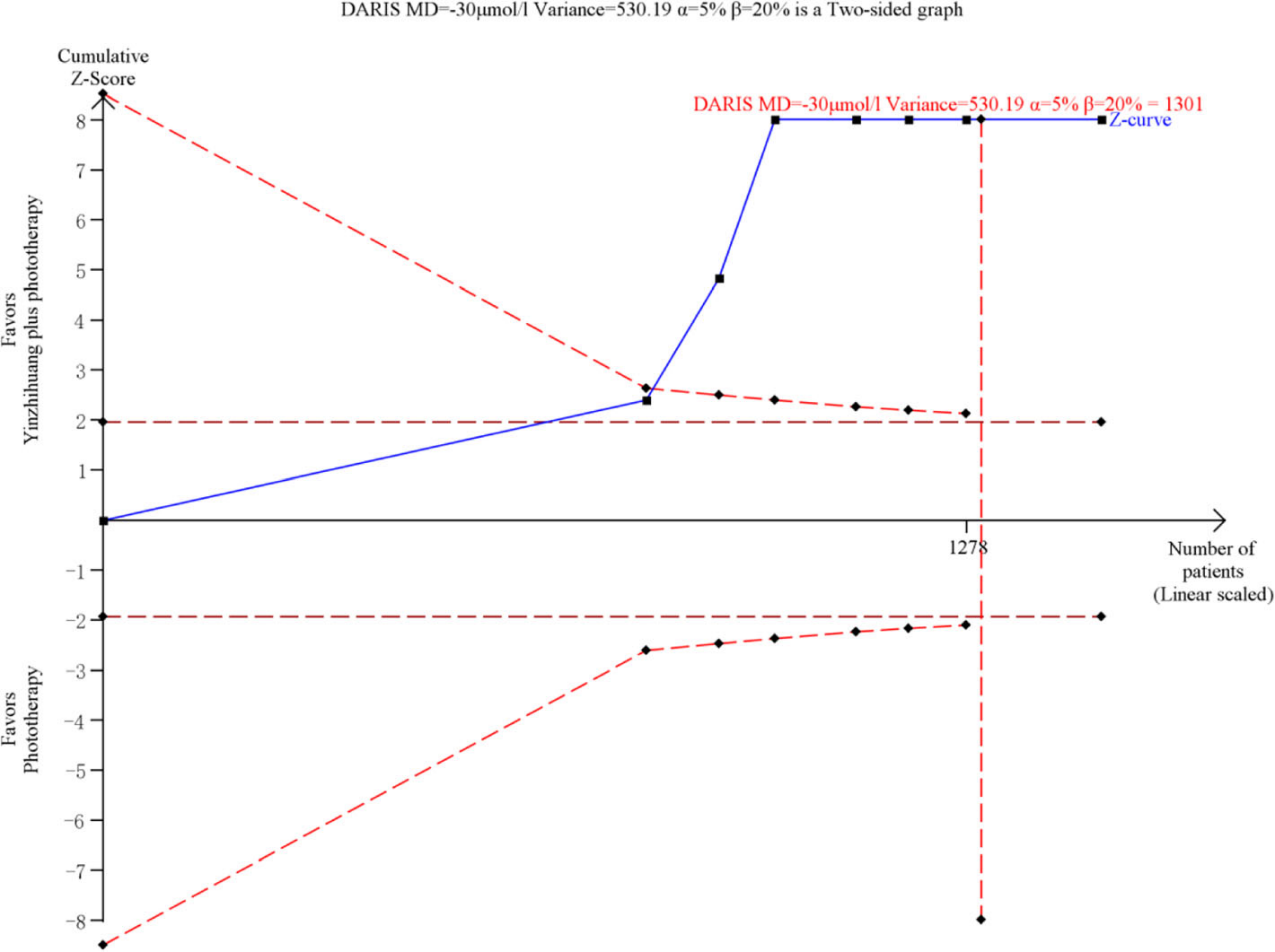
Trial sequential analysis of total serum bilirubin for Yinzhihuang oral liquid combined with phototherapy versus phototherapy monotherapy. Note: Trial sequential analysis illustrated that the cumulative Z-curve (blue curve) had crossed the monitoring boundaries (red inward sloping curves) and reached the diversity-adjusted required information size (DARIS), which is calculated to be 1301 patients (vertical line) based on a mean difference (MD) of -30umol/L, variance of 530.19, type I error of 5% and type II error of 20%.) of 20%, and diversity (D) of 30%

Trial sequential analysis of the total serum bilirubin was based on 7 trials with 1478 participants (Fig. 4). The diversity-adjusted required information size (DARIS) of 1301 participants was calculated on the basis of a type I error of 5%, type II error of 20%, mean difference of serum bilirubin of -30 μmol/L, and variance of 530.19. The cumulative Z-curve has crossed the trial monitoring boundaries as well as the sequential monitoring boundaries and reached the required information size.

##### Serious adverse events

Eight trials (involving 751 participants) provided information on serious adverse events and reported that no participants had serious adverse events during treatment or in the follow-up period.

#### Secondary outcomes

##### Failure of jaundice resolution

**Fig. 5:**
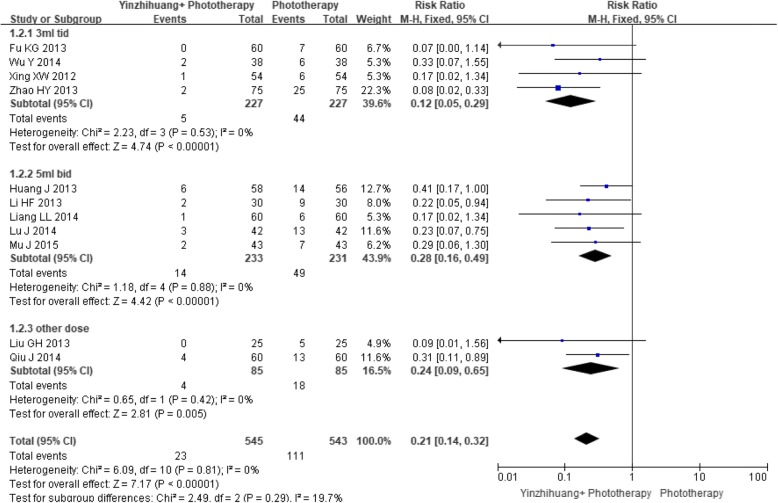
Forest plot of failure of jaundice resolution and subgroup analysis based on dosage of Yinzhihuang oral liquid

Eleven trials provided information on failure of jaundice clearance. In the meta-analysis, 23 of the 545 participants in Yinzhihuang oral liquid combination group did not achieve jaundice resolution versus 111 of the 543 participants in phototherapy monotherapy group. Yinzhihuang oral liquid combined with phototherapy was found to be superior to phototherapy alone for jaundice resolution (RR 0.21, 95% CI 0.14 to 0.32, I^2^ = 0%; 11 trials, *n* = 1088, FEM). Subgroup analysis on dosage revealed that combination therapy was better than phototherapy monotherapy for all doses:3 ml Tid (RR 0.12, 95% CI 0.05 to 0.29, I^2^ = 0%; 4 trials, *n* = 454, FEM), 5 ml Bid (RR 0.28, 95% CI 0.16 to 0.49, I^2^ = 0%; 5 trials, *n* = 464, FEM) and other doses (RR 0.24, 95% CI 0.09 to 0.65, I^2^ = 0%; 2 trials, *n* = 170, FEM) (Fig. 5).

##### Publication bias

**Fig. 6:**
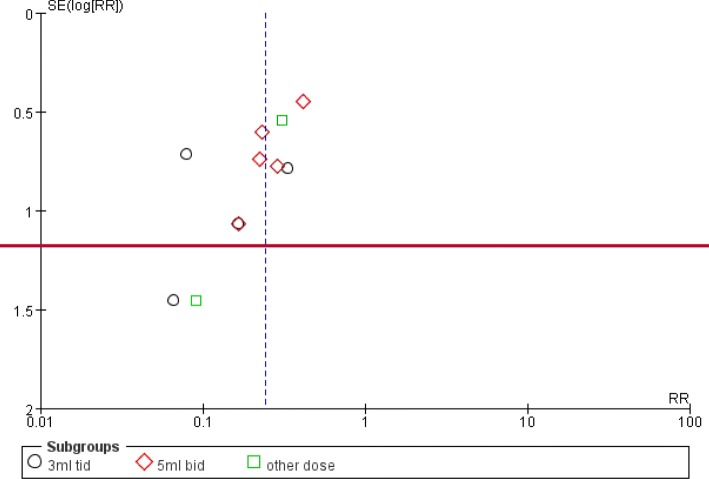
Funnel plot of failure of jaundice resolution

The funnel plot was asymmetric (Fig. 6) suggesting that there may be publication bias.

##### Time to jaundice resolution

**Fig. 7:**
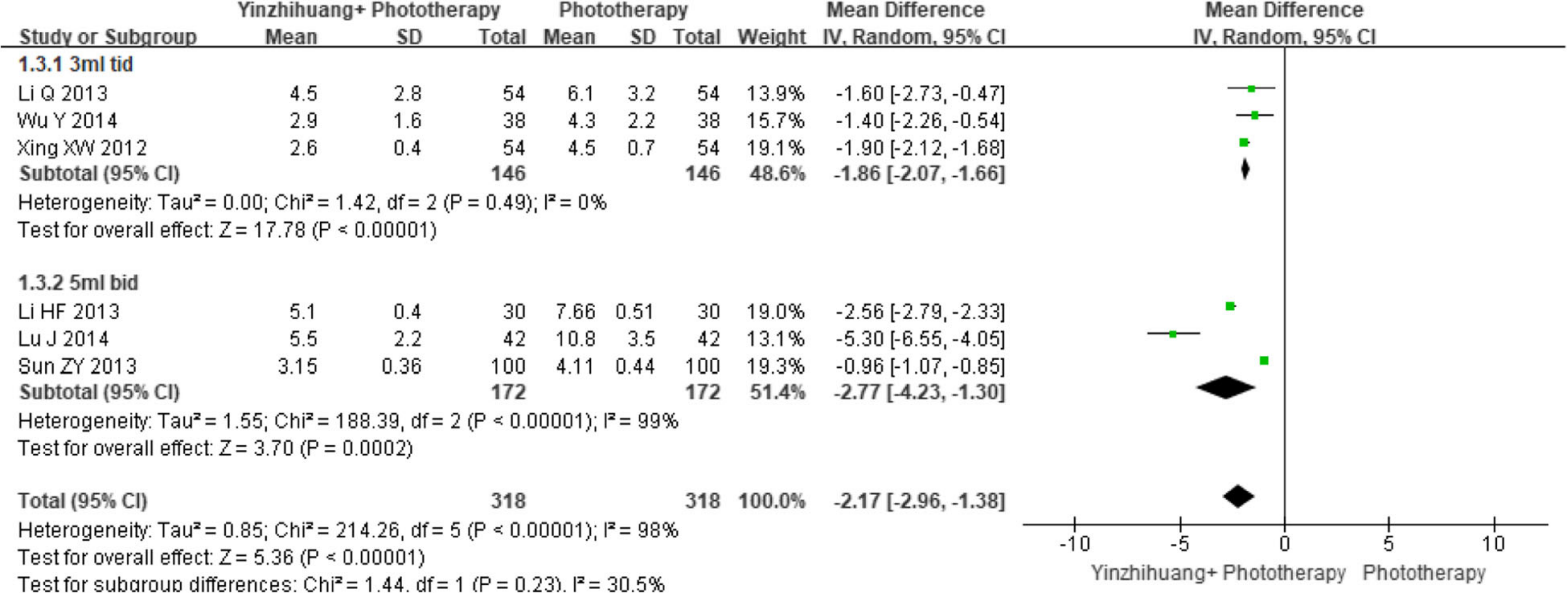
Forest plot of time to jaundice resolution and subgroup analysis based on dosage of Yinzhihuang oral liquid

Six trials provided information on the time to jaundice resolution. The meta-analysis of the 318 patients in Yinzhihuang oral liquid combination group compared with 318patients in phototherapy alone group revealed that the combination therapy was superior to phototherapy alone for reducing the time to jaundice resolution (MD − 2.17 days, 95% CI -2.96 to − 1.38, I^2^ = 98%; 6 trials; *n* = 636, REM). Subgroup analysis based on dosage also revealed that combination therapy was better than phototherapy for 3 ml tid (Fig. 7).

##### Non-serious adverse event

Nine studies reported on adverse events. In five studies no drug-related non-serious adverse events were reported while four studies reported digestive tract symptoms such as diarrhea (39/771 in Yinzhihuang oral liquid combination group and 29/784in phototherapy alone group) or vomiting (22/376 in Yinzhihuang oral liquid combination group and 18/375 in phototherapy monotherapy group) or rash (61/771 in Yinzhihuang oral liquid combination group and 67/784 in phototherapy monotherapy group).

#### Sensitivity analysis

##### Summary of findings

The Grading of Recommendations Assessment, Development, and Evaluation (GRADE) ‘Summary of findings’ table (Guyatt 2008) is shown in Table 2.

**Table 2:** Summary of findings’ table

Outcomes	Illustrative comparative risks* (95% CI)		Relative effect (95% CI)	No of Participants (studies)	Quality of the evidence (GRADE)	Comments
	Assumed risk	Corresponding risk				
	Phototherapy	Yinzhihuang oral liquid combined with Phototherapy				
Total serum bilirubin serological tests Follow-up: 0–2 weeks		The mean total serum bilirubin in the intervention groups was 50.25 μmol/l lower (64.01 to 36.50 lower)		1478 (7 studies)	⊕⊝⊝⊝ very low^a,b^	
Time tojaundice resolution Clinical observation Follow-up: 0–2 weeks		The mean time to jaundice resolution in the intervention groups was 2.17 days lower (2.96 to 1.38 lower)		636 (6 studies)	⊕⊝⊝⊝ very low^a,c^	
Failure of jaundice resolution Clinical observation and serological tests Follow-up: 0–2 weeks	Study population		RR 0.21 (0.14 to 0.32)	1088 (11 studies)	⊕⊝⊝⊝ very low^a,d^	
	204 per 1000	43 per 1000 (29 to 65)				
	Medium risk population					
	200 per 1000	42 per 1000 (28 to 64)				

Yinzhihuang oral liquid combined with Phototherapy compared to Phototherapy alone for neonatal jaundice

Patient or population: patients with neonatal jaundice

Intervention: Yinzhihuang oral liquid combined with Phototherapy

Comparison: Phototherapy alone

*The basis for the assumed risk (e.g. the median control group risk across studies) is provided in footnotes. The corresponding risk (and its 95% confidence interval) is based on the assumed risk in the comparison group and the relative effect of the intervention (and its 95% CI)

CI: Confidence interval; RR: Risk ratio

GRADE Working Group grades of evidence

High quality: Further research is very unlikely to change our confidence in the estimate of effect

Moderate quality: Further research is likely to have an important impact on our confidence in the estimate of effect and may change the estimate

Low quality: Further research is very likely to have an important impact on our confidence in the estimate of effect and is likely to change the estimate

Very low quality: We are very uncertain about the estimate

^a^Most of the included studies were assessed as high risk or unclear risk

^b^Heterogeneity could be detected with the Value of I squire is 98%

^c^Heterogeneity could be detected with the Value of I squire is 98%

^d^Clinical heterogeneity might exist for different dosage and baseline characteristics of participants

## Discussion

### Summary of the main finding

This study focuses on evaluating the effectiveness and safety of Yinzhihuang oral liquid in combination of phototherapy for treating neonatal jaundice compared with phototherapy alone. Seventeen RCTs were included and most of them were assessed as having a high risk of bias. Only seven of the included studies reported our primary outcome of serum bilirubin levels. All the included trials had focused on the effect of this herbal medicine on jaundice resolution but only nine of them reported on the presence or absence of side effects. Based on the performed meta-analysis, Yinzhihuang oral liquid combined with phototherapy appears to be safe and to be superior to conventional phototherapy monotherapy, especially for reducing serum bilirubin, the meta-analysis was supported by trial sequential analysis, suggesting that the required sample size had been reached and the results are unlikely to be influenced by type I error. The combination therapy may also be superior to phototherapy monotherapy in reducing the time required for jaundice resolution.

### Compared with previous studies

There have been two systematic reviews [36, 37] on the use of Traditional Chinese Medicine for neonatal jaundice, which were published in Chinese. Both suggested a benefit for using a combination of Chinese and Western medicine (Phenobarbital, probiotics or phototherapy), compared with Western medicine, for the treatment of neonatal jaundice. However, these two reviews were only interested in the “response rate”, which is a composite outcome that merged different types of outcomes, which is likely to result in the increased probability of type I error. Moreover, these two systematic reviews did not separate interventions by subgroup analysis according to different types of comparisons (Phenobarbital, probiotics or phototherapy in the control group). Our research has focused on the comparison between Yinzhihuang oral liquid and phototherapy versus phototherapy monotherapy, and defined the outcomes more accurately and preciously. Our study suggests that Yinzhihuang oral liquid has a synergistic effect on phototherapy in reducing serum bilirubin, attaining jaundice resolution and shortening the time to jaundice resolution, which is similar to findings in the previous studies.

According to TCM theory, it is believed that neonatal jaundice is related to innate factors (born with this defect), and it’s main syndrome in TCM (used to describe different condition of the same disease) are fetal jaundice with the syndrome of stagnation and steaming of damp-heat, internal retention of cold wetness or syndrome of qi stagnation and blood stasis. Yinzhihuang oral liquid is composed of *Herba Artemisiae Scopariae* extraction, *Fructus Gardeniae* extraction, *Radix Scutellariae* extraction and a small amount of *Flos Lonicerae Japonicae* extraction. The oral liquid is mainly used to treat jaundice caused by retention of damp-heat in the interior. The Chinese herb *Herba Artemisiae Scopariae* has the function of clearing heat and removing toxicity and normalizing gallbladder function to treat jaundice. Western medicine has proved that the ethanol extract of *Herba Artemisiae Scopariae* can induce rats’ mitochondria [38] to protect against drug-induced liver damage.

### Overall completeness and applicability

Based on the poor quality of the included studies, the overall grade of the evidence is low. We only identified 17 eligible trials in this review with a high degree of heterogeneity and publication bias between studies. For the pooled data of total serum bilirubin, we conducted subgroup analysis based on different dosages of intervention herbal medicine. However, heterogeneity still remained and I^2^ values were beyond 75%. In the original studies, baseline characteristics of participants and information about the drug manufacturers were rarely presented. However, we did find different drug sources of Yinzhihuang oral liquid (produced in different pharmaceutical companies) across the studies, which might explain the existed heterogeneity. Even though we synthesized the data in the meta-analysis, we could not be absolutely certain about the positive effect of Yinzhihuang oral liquid. Yinzhihuang oral liquid appears to be safe and have a synergistic effect on phototherapy for baby with neonatal jaundice, and may be a promising adjunct and complementary therapy in clinical practice. Suggested doses for newborns are 3 ml tid or 5 ml bid.

### Implications for further studies

None of the included studies were large scale multi-centred trials. The methodological quality of included trials was low. When compared with phototherapy, Yinzhihuang plus phototherapy showed a greater effect on jaundice resolution, which was also supported by trial sequential analysis (Fig. 4). We suggest conducting further studies of phototherapy plus Yinzhihuang compared with phototherapy plus placebo. Particular attention should focus on the different effects on jaundiced neonates with different levels of serum bilirubin to determine whether this treatment could be used for high risk neonates. If possible, this herbal medicine should be tested on babies of different ethnicity. The safety and efficacy of this treatment needs to be confirmed and the dosage range also needs to be determined.

## Conclusion

Based on 17 trials with low methodological quality, Yinzhihuang oral liquid combined with phototherapy seemed to be safe and more effective than phototherapy alone for reducing serum bilirubin in jaundiced neonates. This observation may be real or caused by systematic errors (methodological quality), but it does not seem to be caused by random error (play of chance). However, due to the limitations of the quality of evidence, we fail to make a conclusive recommendation about Yinzhihuang oral liquid plus phototherapy for treating neonatal jaundice. We suggest conducting rigorously designed RCT for Yinzhihuang oral liquid on neonates with high levels of serum bilirubin, to avoid systematic errors.

## Appendix

**Table 3:** Specific search strategies

Database	Time span	Specific search strategy	Number of hit
Medline	Inception to December 2017	(Yinzhihuang * or Yinchenhao decoction * or Herba Artemisiae Scopariae).mp. [mp = title, abstract, original title, name of substance word, subject heading word, keyword heading word, protocol supplementary concept word, rare disease supplementary concept word, unique identifier]	57
Cochrane Library	Issue12, 2017	Yinzhihuang:ti,ab,kw or Yinchenhao decoction:ti,ab,kw or Herba Artemisiae Scopariae:ti,ab,kw (Word variations have been searched)	136
ClinicalTrials.gov	Inception to December 2017	Drop Interventions clause leaving: Yinzhihuang or Yinchenhao decoction or Herba Artemisiae Scopariae	1
WHO ICTRP	Inception to December 2017	“Yinzhihuang OR Yinchenhao decoction OR Herba Artemisiae Scopariae” in the Intervention	1
CNKI	Inception to December 2017	((Title/Abstract/Keyword = Artemisiae AND Title/Abstract/Keyword = Scopariae) OR Title/Abstract/Keyword = Yinzhihuang+Yinchenhao+Yinchen+Yinchenhaotang) AND Title/Abstract/Keyword = Xinshenger+Neonatal+Neonate+Newborn+Babies AND Title/Abstract/Keyword = Huangdan+Gaodanhongsu+hyperbilirubinemia AND Title/Abstract/Keyword = Suiji+Random+Randomisation	572
VIP	Inception to December 2017	(((Title/Keyword = Artemisiae OR Abstract = Artemisiae) AND (Title/Keyword = Scopariae OR Abstract = Scopariae)) OR Title/Keyword = Yinzhihuang OR Title/Keyword = Yinchenhao OR Title/Keyword = Yinchen OR Title/Keyword = YinchenhaotangOR Abstract = Yinzhihuang OR Abstract = Yinchenhao OR Abstract = Yinchen OR Abstract = Yinchenhaotang) AND (Title/Keyword = Xinshenger OR Title/Keyword = Neonatal OR Title/Keyword = Neonate OR Title/Keyword = Newborn OR Title/Keyword = Babies OR Abstract = Xinshenger OR Abstract = Neonatal OR Abstract = Neonate OR Abstract = Newborn OR Abstract = Babies) AND (Title/Keyword = Huangdan OR Title/Keyword = Gaodanhongsu OR Title/Keyword = hyperbilirubinemia OR Abstract = Huangdan OR Abstract = Gaodanhongsu OR Abstract = hyperbilirubinemia) AND (Title/Keyword = Suiji OR Title/Keyword = Random OR Title/Keyword = Randomisation OR Abstract = Suiji OR Abstract = Random OR Abstract = Randomisation)	616
Wanfang	Inception to December 2017	(((Title/Abstract/Keyword = Artemisiae)*(Title/Abstract/Keyword = Scopariae)) + (Title/Abstract/Keyword = Yinzhihuang+Yinchenhao+Yinchen+Yinchenhaotang))*(Title/Abstract/Keyword = Xinshenger+Neonatal+Neonate+Newborn+Babies)*(Title/Abstract/Keyword = Huangdan+Gaodanhongsu+hyperbilirubinemia)*(Title/Abstract/Keyword = Suiji+Random+Randomisation)	692
Sinomed	Inception to December 2017	#1 (((((“Huangdan”[Title]) OR “Gaodanhongsu”[Title]) OR “hyperbilirubinemia”[Title]) OR “Huangdan”[Abstract]) OR “Gaodanhongsu”[Abstract]) OR “hyperbilirubinemia”[Abstract] #2 (((((((((“Xinshenger”[Title]) OR “Neonatal”[Title]) OR “Neonate”[Title]) OR “Newborn”[Title]) OR “Babies”[Title]) OR “Xinshenger”[Abstract]) OR “Neonatal”[Abstract]) OR “Neonate”[Abstract]) OR “Newborn”[Abstract]) OR “Babies”[Abstract] #3 (((((((“Yinzhihuang”[Title]) OR “Yinchenhao”[Title]) OR “Yinchen”[Title]) OR “Yinchenhaotang”[Title]) OR “Yinzhihuang”[Abstract]) OR “Yinchenhao”[Abstract]) OR “Yinchen”[Abstract]) OR “Yinchenhaotang”[Abstract]) #4 (((((“Suiji”[Title]) OR “Random”[Title]) OR “Randomisation”[Title]) OR “Suiji”[Abstract]) OR “Random”[Abstract]) OR “Randomisation”[Abstract] #5 (#4) AND (#3) AND (#2) AND (#1)	553

## Abbreviations

AAP: American Academy of Pediatrics
Bid: Twice a day
CI: Confidential Interval
CNKI: China Network Knowledge Infrastructure
DARIS: Diversity-adjusted Required Information Size
FEM: Fixed Effect Model
GRADE: The Grading of Recommendations Assessment, Development, and Evaluation
MD: Mean Difference
NA: Not applicable
Po: Peroral
PRISMA: Preferred Reporting Items for Systematic Review and Meta-analysis
RCT: Random Controlled Trial
REM: Random Effects Model
RR: Relative Risk
Sino-Med: Chinese Biomedical Literature Database
TBil: Total bilirubin
TCB: Transcutaneous Bilirubin
TCM: Traditional Chinese Medicine
Tid: Three times a day
VIP: Chinese Scientific Journal Database
WHO ICTRP: World Health Organization Clinical Trials Registry Platform
